# Relation of 91 Circulating Inflammatory Proteins to Nonalcoholic Fatty Liver Disease: A Two‐Sample Mendelian Randomisation Study

**DOI:** 10.1111/jcmm.70322

**Published:** 2024-12-25

**Authors:** Xiaodong Wu, Yanhong Song, Shuodong Wu

**Affiliations:** ^1^ Department of General Surgery Shengjing Hospital of China Medical University Shenyang China; ^2^ Department of Anesthesiology Shengjing Hospital of China Medical University Shenyang China

**Keywords:** causal effect, genetic prediction, inflammatory proteins, Mendelian randomisation study, NAFLD

## Abstract

Increasingly, emerging research evidence has demonstrated that nonalcoholic fatty liver disease (NAFLD) is a disease closely associated with systemic inflammation. However, the specific upstream inflammatory factors engaged in the pathogenesis of NAFLD remain unclear. Our study aimed to identify the inflammatory regulators causally associated with NAFLD pathogenesis through Mendelian randomisation. A two‐sample Mendelian randomisation method was applied to analyse the causal association between 91 circulating inflammatory proteins and NAFLD. Data on circulating inflammatory proteins were derived from samples of European ancestry (14,824 samples) and NAFLD data were obtained from the FinnGen consortium (2025 cases and 284,826 controls). Instrumental variables were selected from the genetic variance and F‐statistics were calculated to avoid bias. We adopted the random‐effects inverse variance weighting (IVW) method as our primary analytical approach. Supplementary analyses were also implemented, including weighted median, MR‐Egger and weighted mode. Moreover, we conducted pleiotropy and heterogeneity analyses to validate the accuracy of the findings. The application of Mendelian randomisation analysis identified four inflammatory factors that might be causally associated with NAFLD at the genetic level. Elevated levels of eotaxin (or = 1.27, 95% CI: 1.05–1.53, *p* = 0.014), osteoprotegerin (OPG) (or = 1.29, 1.03–1.60, *p* = 0.023) and TNFRSF9 (or = 1.32, 95% CI: 1.06–1.64, *p* = 0.014) may be causally related to an increasing risk of NAFLD. Conversely, heightened leukaemia inhibitory factor (LIF) levels (or = 0.63, 0.44–0.92, *p* = 0.016) were linked to a lower risk of NAFLD onset. There was no causal relationship between levels of other circulating inflammatory proteins and NAFLD. Our analysis uncovered four upstream inflammatory factors genetically associated with the pathogenesis of NAFLD. These results highlight the potential involvement of inflammation in NAFLD, which provides partial insights for further research in this field in the future.

## 1 Introduction

Nonalcoholic fatty liver disease (NAFLD) has become a major health concern globally, and it is strongly associated with the increasing prevalence of metabolic syndrome, obesity and type 2 diabetes [1]. According to epidemiologic analysis, its global prevalence has reached 38% [2]. It represents a spectrum of liver diseases ranging from simple steatosis to nonalcoholic steatohepatitis (NASH) and, in severe cases, progressing to cirrhosis and hepatocellular carcinoma [3]. Although previous studies have concluded that obesity is an essential causative factor for NAFLD, nonobese NAFLD still represents a significant portion of the global population [4], implying that the underlying mechanisms and causative factors contributing to the emergence and progression of NAFLD remain complicated and multifaceted.

Several studies have explored the potential link between systemic inflammation and NAFLD, postulating that various cytokines and growth factors might exert a pivotal effect on the disease's pathogenesis [5, 6, 7]. Inflammation is one of the hallmarks of NAFLD, with studies implicating the imbalance of anti‐inflammatory and proinflammatory cytokines in the onset and progression of liver steatosis [8, 9, 10]. However, the existing literature presents a landscape of inconsistent findings, leaving unresolved questions regarding the precise relationship between systemic inflammation and NAFLD.

The debate surrounding the association between inflammatory markers and NAFLD has been characterised by methodological limitations inherent in observational studies. Issues such as confounding, reverse causation and unmeasured biases have contributed to the discrepancies observed across different investigations [11]. To address these challenges and elucidate the causal relationship between systemic inflammation and NAFLD, our study employs a robust Mendelian randomisation (MR) approach, offering a powerful instrument to deduce causality by leveraging genetic variance as proxies for modifiable exposures [12, 13]. By exploiting genetic instruments and large‐scale datasets, we aim to get over the limitations of traditional observational studies and provide more reliable insights into the causal role of inflammatory markers in NAFLD. In the present study, we retrieved 91 inflammatory markers and NAFLD from the publicly available GWAS database to explore the causal relationship between inflammatory markers and NAFLD with a two‐sample MR analysis. In summary, this study seeks to contribute valuable insights to comprehend the intricate association between systemic inflammatory markers and NAFLD, utilising the precision and robustness afforded by Mendelian randomisation.

## 2 Materials and Methods

### 2.1 Research Design

**FIGURE 1:**
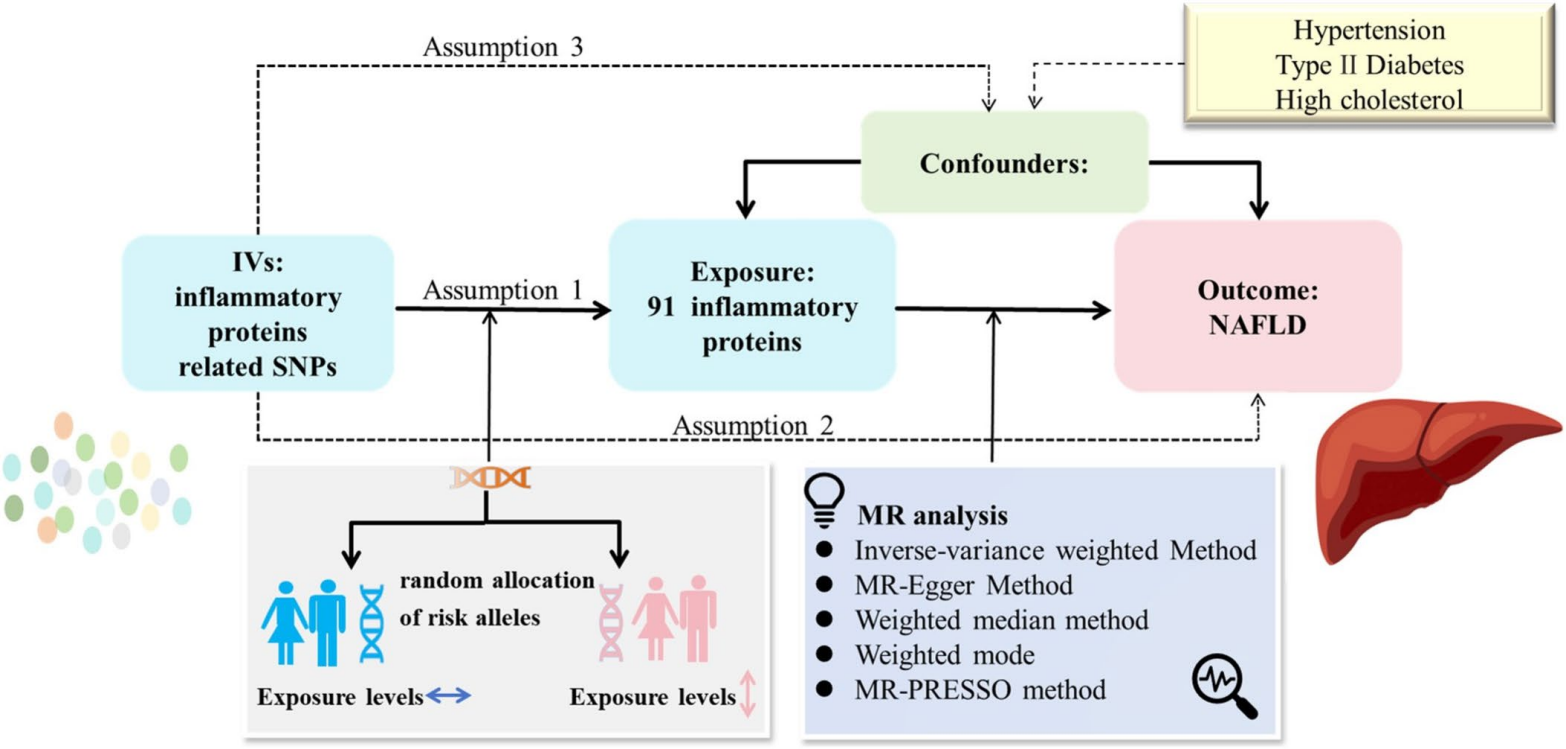
Study design overview.

A brief illustration of the design of our study is presented in Figure 1. This study was conducted under the guidance of the STROBE‐MR guidelines [14]. We obtained exposure‐ and outcome‐related genetic variants from genome‐wide association studies that are publicly available. All study populations were of European ancestry and there was no sample duplication between the exposure and outcome. The study design rationale was based on three main hypotheses: (1) Genetic prediction was strongly associated with exposure (91 inflammatory proteins) in this study; (2) Genetic prediction only affected outcome through the exposure of interest (91 inflammatory proteins) and not directly on outcome (NAFLD) and (3) Genetic prediction is not associated with potential confounders [15]. All analysed data used in this research article were publicly available online rather than individual‐level data, and therefore no ethics committee approval was required.

### 2.2 Data Source for Inflammatory Proteins and NAFLD

This study was conducted with published large‐scale GWAS studies of circulating concentrations of 91 inflammatory proteins, which included 11 cohorts totalling 14,824 participants of European ancestry [16]. Zhao et al. performed measurements of inflammatory proteins by applying the Olink Target‐96 Inflammation immunoassay panel. The summary‐level statistics on NAFLD were obtained from the Finnish Genetic Consortium's genomic study of 377,277 individuals of European ancestry (version R9) [17]. The FinnGen study is a project intended to assemble and analyse the genomic and wellness data of 500,000 unique samples. The GWAS for NAFLD in the FinnGen study contains 2025 cases and 284,826 controls. In this study, there was no sample overlap between the data sources for inflammatory proteins and NAFLD. Therefore, the risk of bias attributable to overlapping samples was minimised.

### 2.3 Selection of Instrumental Variables

Genetic variants that were both reliably (*p* < 5E‐5) and independently (not in linkage disequilibrium, *r* ^2^ < 0.001 within a 10,000‐kb window) correlated with each inflammatory protein were selected as instrumental variables. Linkage disequilibrium was estimated based on the reference panel of the European 1000 Genomes Project. Potential instrumental variables should be able to be harmonised with the outcome SNPs, and SNPs that are correlated with the outcome (*p* > 5E‐5) need to be eliminated. MR Steiger was applied to filter SNPs with invalid causal directions. The strength of association (F) was calculated for all instrumental variables to avoid the bias of weak instrumental variables (F < 10) on the assessment results. We also used MR Pleiotropy Residual Sum and Outlier (MRPRESSO) to identify potential outliers and remove them. Phenoscanner, a human genotype–phenotype association database containing extensive information, was the site where we checked to remove SNPs associated with confounders. Eventually, all eligible instrumental variables are summarised in Table S1.

### 2.4 MR Analysis

To explore the causal effect of circulating inflammatory proteins on NAFLD, we analysed a total of 91 associations (91 exposures × 1 outcome). The Bonferroni method was utilised to correct for the significance threshold (*p* < 5.5E‐4), while we considered the association between exposure and outcome as nominally significant when the *p*‐value was between 5.5E‐4 and 0.05. Random‐effects inverse variance weighting was adopted as the primary analytic method to assess the causal role of each pair of associations. However, the continued use of IVW methods may lead to biased estimates of causal effects when there are multivalence problems with instrumental variables. MR‐Egger and weighted medians can be helpful in the presence of horizontal multivalence. The results of MR‐Egger are plausible when the share of the instrumental variable for horizontal pleiotropy is more than 50% [18], while the results of the weighted median seem to be more robust when the percentage is less than 50%. The weighted model has also been applied as a complementary analysis method. Horizontal pleiotropy was detected by MR‐PRESSO and MR‐Egger regression. Cochran's *Q* test was used as the main assessment of heterogeneity. We also plotted scatter plots, funnel plots and leave‐one‐out plots, which can be used as a complement to sensitivity analyses. All statistical analysis processes were conducted with R version 4.3.0 and its packages ‘TwoSampleMR’ and ‘MRPRESSO’.

## 3 Results

**FIGURE 2:**
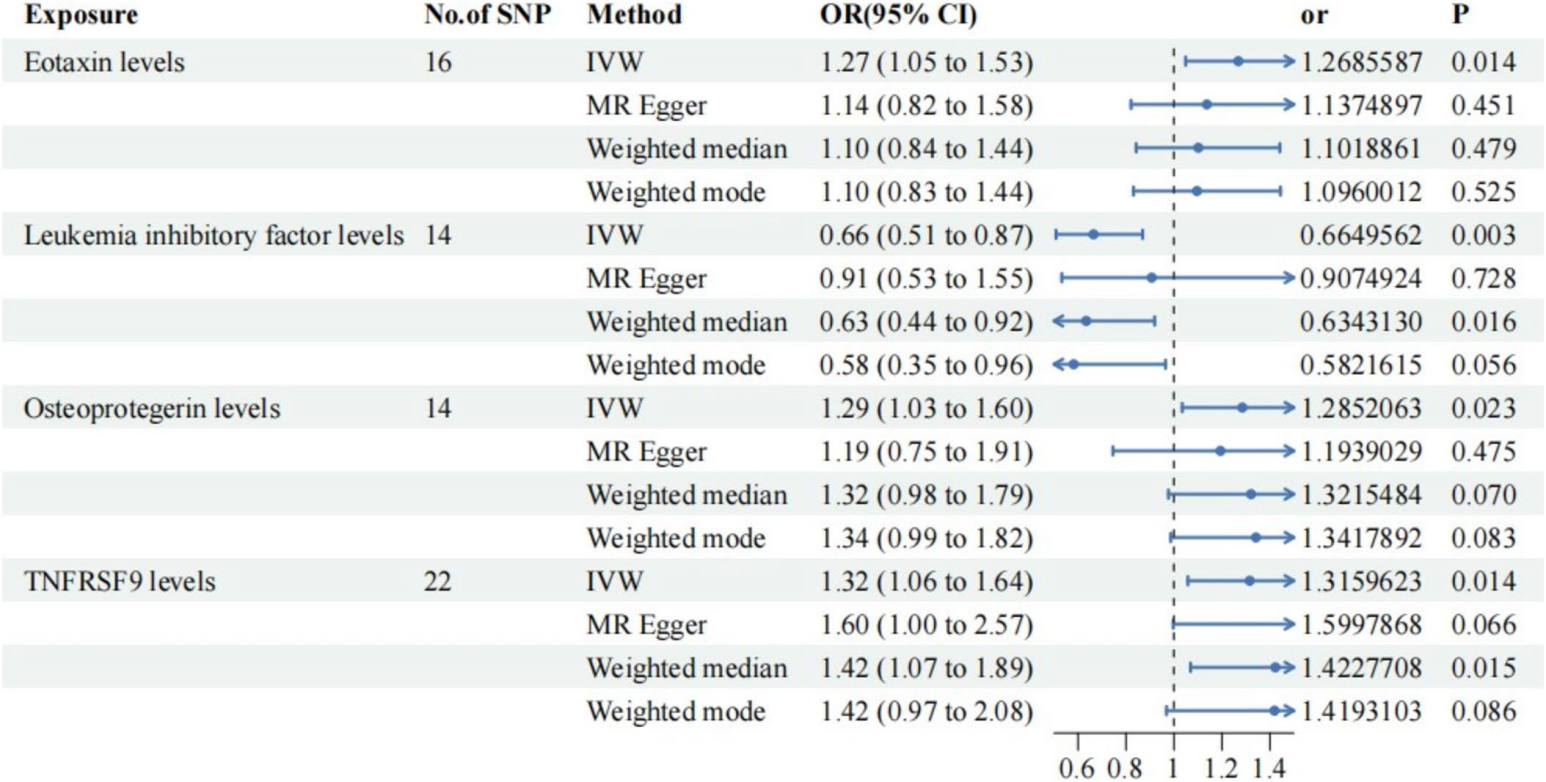
Association of serum eotaxin levels, leukaemia inhibitory factor levels, osteoprotegerin levels and TNFRSF9 levels with risk of NAFLD, analysed with four different Mendelian randomisation (with forest plot).

**FIGURE 3:**
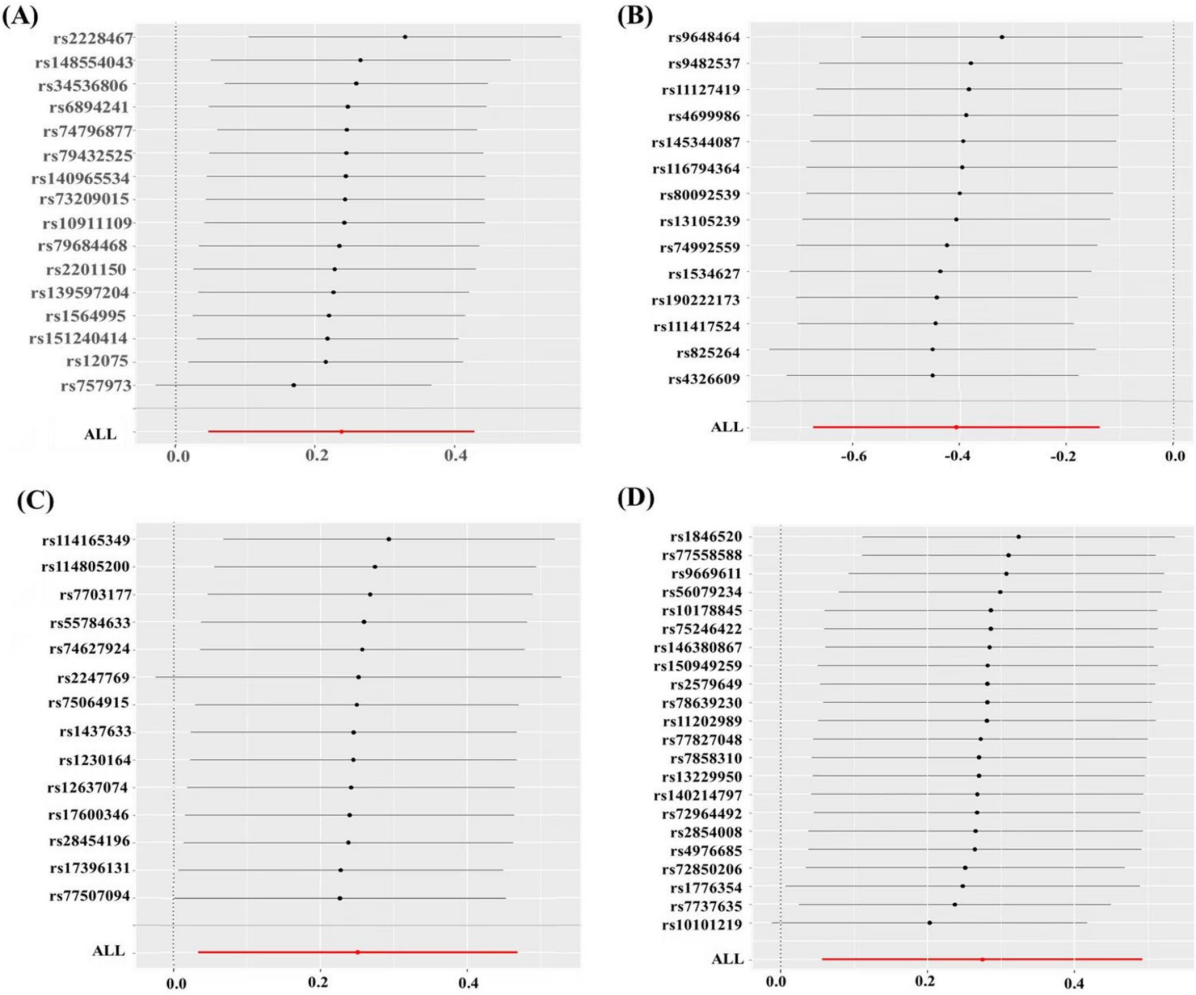
‘Leave one out’ analysis. (A) MR leave‐one‐out sensitivity analysis for serum eotaxin levels on NAFLD. (B) MR leave‐one‐out sensitivity analysis for serum leukaemia inhibitory factor levels on NAFLD. (C) MR leave‐one‐out sensitivity analysis for serum osteoprotegerin levels on NAFLD. (D) MR leave‐one‐out sensitivity analysis for serum TNFRSF9 levels on NAFLD.

To explore the causal relationship between the levels of 91 circulating inflammatory proteins and NAFLD, we included a total of 1706 instrumental variables. The number of SNPs analysed used in all associations ranged from 7 to 34. The F‐statistics for these genetic instruments ranged from 16.89 to 1532.72 indicating the exclusion of weak instrumental variables. Details of these instrumental variables have been illustrated in Table S1. A total of four causal relationships were identified in our study. As shown in Figure 2, The random‐effects IVW results estimated that genetically predicted eotaxin levels (or = 1.27, 95% CI: 1.05–1.53, *p* = 0.014), OPG levels (or = 1.29, 1.03–1.60, *p* = 0.023) and tumour necrosis factor receptor superfamily member 9 (TNFRSF9) levels (or = 1.32, 95% CI: 1.06–1.64, *p* = 0.014) had significant adverse effects on the development of NAFLD, while elevated of LIF levels (or = 0.66, 95% CI: 0.51–0.87, *p* = 0.003) reduce NAFLD onset. Other MR analyses also provide partial support, with the weighted median suggesting that elevated TNFRSF9 levels increased the risk of NAFLD (or = 1.42, 1.07–1.89, *p* = 0.015), while elevated levels of LIF were negatively correlated with the risk of NAFLD (or = 0.63, 0.44–0.92, *p* = 0.016). Further sensitivity tests suggested that there was no heterogeneity or multiplicity in these four causal analyses (*p* > 0.05). As shown in Figure 3, the results of the culling analysis revealed no potential effect of SNPs driving the causal effect between inflammatory proteins and NAFLD. The results of MR analysis and sensitivity analysis regarding other inflammatory proteins with NAFLD are available in Table S2. To verify the causal effect of NAFLD with circulating inflammatory proteins, we performed a reverse MR analysis, which showed no reverse causal effect between NAFLD and all 91 circulating inflammatory proteins. The results of reverse MR are shown in Table S3.

## 4 Discussion

NAFLD is a complex and multifactorial condition with a rising global prevalence, prompting extensive research to unravel its underlying mechanisms and identify potential therapeutic targets [3]. Our Mendelian randomisation study sought to contribute to this growing body of knowledge by investigating the causative link between 91 inflammatory markers and the development of NAFLD. The observed causal associations between inflammatory markers and NAFLD can be contextualised within theoretical frameworks that emphasise the interplay between inflammation, metabolic dysregulation and hepatocellular damage. Chronic low‐grade inflammation is recognised as a central driver in the progressive development from simple steatosis to more severe forms of NAFLD [19]. The identified inflammatory markers may act as mediators or modulators of this inflammatory response, influencing disease progression. The results of our study identified four systemic inflammatory markers—Eotaxin, LIF, OPG and TNFRSF9—with distinct causal effects in the pathogenesis of NAFLD.

The adverse effects of eotaxin, OPG and TNFRSF9 on NAFLD development underscore the potential role of these inflammatory markers in the pathophysiology of hepatic steatosis. Eotaxin is positively correlated with hepatic triglyceride levels [20], and ectopic accumulation of fat may constitute the pathologic basis of NAFLD [21]. Moreover, in a mouse model of immune‐mediated hepatitis, the administration of an oral antieotaxin‐1 antibody demonstrated hepatoprotective and anti‐inflammatory effects [22]. Similarly, our study genetically confirmed a causal link between high levels of eotaxin and the development of NAFLD. Osteoprotegerin, a cytokine associated with bone remodelling, has been implicated in chronic inflammatory conditions and may play a role in the hepatic inflammatory response observed in NAFLD [23]. A mouse‐based animal study confirmed that OPG upregulates CD36 expression and thus promotes hepatic fat accumulation through activation of a cascade reaction, which may partially contribute to the development of NAFLD [24]. Our MR study similarly supported a causal relationship between increased OPG levels and NAFLD progression. Interestingly, patients with NAFLD often exhibit lower OPG levels compared to healthy individuals [25]. A possible interpretation is the compensatory protective response of the body to avoid more fat accumulation. Downregulation of OPG expression or disruption of the link between OPG and downstream pathways may be a promising future therapeutic direction for NAFLD. TNFRSF9 has been linked to various immune and inflammatory processes [26]. 4‐1BB, as a member of the TNF receptor superfamily, is capable of binding to its ligand to regulate inflammatory signalling and functions in diseases such as obesity and fatty liver [27]. TNFRSF9 regulates T‐cell depletion and apoptosis through activation of the 4‐1BB/TRAF1 pathway, which may be implicated in the pathogenesis of liver fibrosis as well as NAFLD [28]. Our study has identified that TNFRSF9 may be genetically connected to NAFLD. In contrast to previous risk factors, our MR analysis revealed that the LIF may be a protective factor for NAFLD. A recently updated study also reached a consistent conclusion that the LIF may prevent hepatic steatosis in patients with NAFLD. Exogenous LIF supplementation appeared to attenuate hepatic steatosis and insulin resistance in a NAFLD mouse model [29]. Considering the carcinogenic effects of LIF, there are limitations in applying it directly as a therapeutic target. In addition, the results of an experiment with knockout animals suggested that animals knocked out of the Kinins B1 receptor existed with lower expression of LIF while exhibiting immunity to NAFLD. Thus the causal relationship between LIF and NAFLD still needs to be verified by a wide range of basic experiments [30].

One of the strengths of our study is the broad range of inflammatory proteins encompassed, and this is the first MR analysis to explore the causal relationship between 91 circulating inflammatory proteins and NAFLD, which has important clinical and public health implications. Specific inflammatory markers causally associated with NAFLD were identified, and modulating the levels or activity of these markers may be a novel strategy for the prevention or treatment of NAFLD, especially in populations at high risk due to obesity, insulin resistance or metabolic syndrome. Second, the genetic variants screened all met the three basic assumptions and were strong instrumental variables. We also performed sensitivity analyses such as heterogeneity and multiple validity tests. The database for the MR analyses was the large‐scale, large‐sample‐size GWAS study, and the method minimised confounding bias to make causal inferences plausible. However, there are some limitations to our research. In some analyses, the quantity of instrumental variables screening out is too few or too homogeneous, which might affect the results of causal inference. Another limitation is that sensitivity analyses may not be possible when the selected instrumental variables are too few. It is worth noting that negative results for certain associations do not necessarily mean that inflammatory proteins do not have a role, and the selection of genetic variants may have caused interference. In addition, we did not consider potential interactions between inflammatory proteins. The participants in this study were of European ancestry, and the applicability of the findings to other ethnic populations remains to be confirmed. The survey data for the study were obtained from large GWAS studies and lacked demographic information under specific phenotypic stratification for subgroup analysis.

Our findings open avenues for further research to deepen our understanding of the complex interplay between systemic inflammation and NAFLD. Future studies could explore the molecular mechanisms through which the identified inflammatory markers exert their effects on hepatic steatosis. Investigating the potential interactions between genetic variants, inflammatory markers and environmental factors may provide additional insights into individual susceptibility to NAFLD.

## 5 Conclusion

Elevated levels of eotaxin, OPG and TNFRSF9 were identified as causally associated with heightened susceptibility to NAFLD, while elevated LIF levels demonstrated a protective effect against NAFLD onset. These findings provide novel insights into the intricate relationship between systemic inflammation and the phenotypes associated with NAFLD.

## Author Contributions

**Xiaodong Wu:** conceptualization (lead), data curation (lead), formal analysis (lead), funding acquisition (lead), investigation (lead), methodology (lead), project administration (lead), resources (lead), software (lead), software (lead), supervision (lead), supervision (lead), validation (lead), validation (lead), visualization (lead), visualization (lead), writing – original draft (lead), writing – original draft (lead), writing – review and editing (lead), writing – review and editing (lead). **Yanhong Song:** conceptualization (supporting), data curation (supporting), formal analysis (supporting), funding acquisition (supporting), investigation (supporting), methodology (supporting), project administration (supporting). **Shuodong Wu:** resources (supporting), software (supporting), supervision (supporting), validation (supporting), visualization (supporting).

## Conflicts of Interest

The authors declare no conflicts of interest.

## Supporting information


**Table S1.** Details of all eligible instrumental variables for 91 circulating inflammatory proteins.

**Table S2.** Verification of causal relationships between 91 circulating inflammatory proteins and NAFLD by four MR statistical methods.

**Table S3.** Inverse MR results between NAFLD and 91 circulating inflammatory proteins.

## Data Availability

The data used in this study were obtained from publicly available GWAS studies, and relevant information can be found in the original article or in the supporting information. You may contact the corresponding author with any other reasonable requests.
